# Association between cardiovascular autonomic neuropathy and left ventricular hypertrophy in young patients with congenital generalized lipodystrophy

**DOI:** 10.1186/s13098-019-0444-8

**Published:** 2019-07-01

**Authors:** Clarisse Mourão Melo Ponte, Virgínia Oliveira Fernandes, Christiane Bezerra Rocha Liberato, Ana Paula Dias Rangel Montenegro, Lívia Aline Batista, Maria Helane Costa Gurgel, Lia Beatriz de Azevedo Karbage, Izabella Tamira Galdino Farias Vasconcelos, Catarina Brasil d’Alva, Renan Magalhães Montenegro Júnior

**Affiliations:** Brazilian Group for the Study of Inherited and Acquired Lipodystrophies, Faculdade de Medicina, Universidade Federal do Ceará, Rua Professor Costa Mendes 1608, Fortaleza, Ceará 60416-200 Brazil

**Keywords:** Autonomic dysfunction, Ventricular hypertrophy, Cardiac disease, Lipodystrophy, Congenital generalized lipodystrophy

## Abstract

**Background:**

Congenital generalized lipodystrophy (CGL) is a rare disorder characterized by the absence of subcutaneous adipose tissue, severe insulin resistance, diabetes mellitus, and cardiovascular complications, including cardiac autonomic neuropathy (CAN), left ventricular hypertrophy (LVH), and atherosclerosis. The present study aimed to access the association between CAN parameters and cardiovascular abnormalities in CGL patients.

**Methods:**

A cross-sectional study was conducted with 10 CGL patients and 20 healthy controls matched for age, sex, BMI, and pubertal stage. We evaluated clinical, laboratory, and cardiovascular parameters—left ventricular mass index (LVMI), interventricular septum thickness (IVS), systolic and diastolic function determined by two-dimensional transthoracic echocardiography; carotid intimal media thickness (cIMT); and cQT interval. Heart rate variability (HRV) was evaluated by spectral analysis components—high frequency (HF), low frequency (LF), very low frequency (VLF), LF/HF ratio, and total amplitude spectrum (TAS)—and cardiovascular reflexes tests (postural hypotension test, respiratory, orthostatic and Valsalva coefficients).

**Results:**

In CGL group, four patients (40%) had LVH and diastolic dysfunction. HF component (parasympathetic control) was lower in LVH patients. CGL patients presented higher values of cIMT and cQT interval than heathy subjects. Inverse association between LVMI and LF (p = 0.011), IVS and LF (p = 0.007), and cIMT and leptin (p < 0.001) were observed, even after adjustments by HOMA-IR, A1c, and blood pressure. In CGL group, there were associations between LMVI and HF component (IC95%: − 1.000; − 00.553), LVMI and TAS (IC95%: − 1.000; − 0.012), and IVS and HF component (IC95%: − 1.000; − 0.371).

**Conclusion:**

The association between increased LV mass and parameters of HRV provides possible speculations about the involvement of CAN in the pathophysiology of the cardiac complications, including LVH, in patients with CGL.

**Electronic supplementary material:**

The online version of this article (10.1186/s13098-019-0444-8) contains supplementary material, which is available to authorized users.

## Background

Congenital generalized lipodystrophy (CGL) is a rare disease of autonomic recessive inheritance with an estimated prevalence of 0.2 a 1.0 cases/million [1]. There are approximately 500 cases described worldwide, with 100 cases described in Brazil [2]. This disease is characterized by the absence of subcutaneous adipose tissue, leptin deficiency, deposition of ectopic fat due to impairs of the metabolic activity and storage capacity of the subcutaneous adipose tissue, hypertriglyceridemia, insulin resistance and a poorly controlled diabetes mellitus (DM) [2].

In addition to metabolic disorders, cardiac abnormalities have also been described in patients with CGL, especially left ventricular hypertrophy (LVH), left ventricular systolic and diastolic dysfunction, systemic arterial hypertension, QT interval enlargement, cardiac arrhythmias and atherosclerosis [3]. Some cases of hypertrophic cardiomyopathy also were previously described [4]. Among the potential mechanisms involved in the development of these cardiac changes are insulin resistance and myocardial accumulation of triglycerides [5]. However, the pathophysiology of cardiomyopathy observed in CGL is not entirely elucidated.

We previously reported that CGL patients present early microvascular complications, including cardiovascular autonomic neuropathy (CAN) [6]. Several studies have observed an association between CAN and myocardial dysfunction in patients with DM, including LVH and diastolic dysfunction, which may occur even in the absence of coronary artery disease (CAD) and hypertension [7].

These observations allow us to speculate that CAN could be a potential mechanism associated with the early development of LVH in patients exposed to severe metabolic abnormalities early in life. Thus, the present study aimed to evaluate the association between cardiovascular abnormalities and CAN parameters in CGL patients.

## Methods

This study is an additional analysis of the association of CAN (previously published [6]) and cardiovascular parameters in young patients of our CGL cohort.

As previously described [6], CAN was evaluated by heart rate variability (HRV) tests, using cardiovascular autonomic reflex tests—Valsalva maneuver (VAL), respiratory (E/I), orthostatic (30/15), and postural hypotension test—and spectral analysis of the HRV, including very low (VLF), low (LF), high (HF) frequency components, LF/HF ratio, and total amplitude spectrum (TAS). CAN tests were performed following the protocols recommended by Spallone et al. [7] (Additional file 1). Clinical, biochemical and CAN parameters of the CGL patients and healthy individuals were shown in Additional file 2.

In the same period of time, we also evaluated cardiovascular parameters, including left ventricular mass index (LVMI), interventricular septum thickness (IVS), systolic and diastolic function, carotid intimal media thickness (cIMT), and cQT interval in the same 10 CGL patients and 20 healthy individuals matched for age, gender, BMI and pubertal stage.

Subsequently, the association between cardiovascular parameters and CAN tests was analyzed.

This study was approved by the ethics committee of the Federal University of Ceará, and written informed consents were obtained from all participants or their parents before inclusion.

### Transthoracic echocardiography

Two-dimensional transthoracic echocardiography was performed in a Vivid7^®^ device (GE Vingmed, System VII, Horton, Norway) by a single echocardiographer. The usual cuts were performed to allow complete study by M-mode, two-dimensional and Doppler techniques (pulsatile, continuous, color and tissue), following the recommendations of the American Society of Echocardiography [8].

For the calculation of the left ventricular mass, measurements were made of the M mode guided by the two-dimensional mode. Measurements of left ventricle (LV) dimensions were made at the end of diastole and the end of systole according to the recommendations cited. Measurements were: final LV diastolic diameter, the diastolic thickness of the IVS and diastolic thickness of the LV posterior wall. The examiner obtained an average of five or more measurements for each parameter per patient. In adults, the presence of LVH was considered when LVMI was > 95 g/m^2^ in women and > 115 g/m^2^ in men [8]. In children and adolescents, the LVH was considered when the LVMI was > 51 g/m^2.7^ [9, 10]. For the classification in concentric and eccentric LVH, the measurement of the relative thickness of the posterior wall of LV (RPLV) was calculated using the formula: (2 × LV posterior wall)/LV diastolic diameter. Concentric LVH was considered when RPLV ≥ 0.42 mm and eccentric LV when RPLV < 0.42 mm. The systolic function was evaluated by measuring the ejection fraction (EF) calculated by the Teichholz formula and considered normal if greater than or equal to 55% [11]. LV diastolic dysfunction was defined according to the Recommendations for the Evaluation of Left Ventricular Diastolic Function by Echocardiography: An Update from the American Society of Echocardiography and the European Association of Cardiovascular Imaging [12].

### Carotid intimal mean thickness and Doppler sonography

The Vivid7^®^ device (GE Vingmed, System VII, Horton, Norway) was used to assess blood flow and determine the cIMT. The cIMT measurement was performed by two-dimensional 2 cm before the carotid bulb, averaging five measurements (the device did not have the automatic measurement). Doppler was used to studying the internal and external common carotid artery flow bilaterally. A single echocardiographer performed the examinations.

### Corrected QT interval

A 12-lead electrocardiographic record was performed to calculate the QT interval, which was measured from the beginning of the QRS complex until the end of the T wave in the D2 lead. For the correction of the QT interval by HR and obtaining the cQT interval, the Bazzet formula was used: QT/√RR [13]. It was considered a prolonged cQT interval > 0.44 s in women and > 0.42 s in men [14].

### Statistical analysis

Data were analyzed using the Statistical Package of Social Science (SPSS Inc., Chicago, IL, USA), version 15.0 for Windows and R3.3.1 software. Continuous variables were described as the mean and median (minimum; maximum), and categorical variables were described according to the relative and absolute frequencies. The Mann–Whitney test was used for continuous variables. Associations between categorical variables were analyzed using the Chi square test and Fisher’s exact test. For the correlation analysis, the correlation Spearman test was used. Generalized linear models with adjustments for potentially confounding variables were used. The Bootstrap technique was used for the estimation of the confidence interval of the Spearman coefficient for the analysis of the CGL group. A *p* value of less than 0.05 was considered statistically significant.

## Results

In CGL group, median age was 12 years (7–30) and six patients were female (60%). Four patients (40%) had LVH and diastolic dysfunction—one case of moderate concentric LVH and three of mild concentric LVH. No patient had systolic dysfunction. There was no patient with hypertrophic cardiomyopathy or asymmetric septal hypertrophy. An individualized description of CGL cases with a summary of the genetic, metabolic and cardiovascular characteristics is shown in Table 1.

**Table 1:** Summary of genotypic, metabolic and cardiovascular data of patients with CGL (n = 10) (Adapted from Ponte et al. [6])

Case Gender/age Pubertal stage	Subtype mutation (gene)	Comorbidities, microvascular complications	Drugs	LVMI (g/m^2.7^) cIMT (mm) cQT (s)^a^	CAN LVH
1 ♀, 7 years M2P2	Type 2 CGL *BSCL2*	High HOMA-IR score, ↓HDL, ↑TG, nephropathy (moderate albuminuria), ↑BP	MTF	63.9 0.61 0.43	Clinical LVH
2 ♀, 7 years M2P2	Type 1 CGL *AGPAT2*	High HOMA-IR score, ↓HDL, ↑TG	None	32.8 0.66 0.45^a^	Absent No LVH
3 ♂, 9 years G1P1	DNA Not available	High HOMA-IR score, ↓HDL, ↑TG	None	36.2 0.57 0.41	Absent No LVH
4 ♀, 10 years M2P2	Type 2 CGL *BSCL2*	DM, high HOMA-IR score, ↓HDL, ↑TG	MTF	44.5 0.53 0.41	Clinical No LVH
5 ♂, 10 years G2P1	Type 2 CGL *BSCL2*	DM, ↓HDL, ↑TG, ↑cholesterol, nephropathy (severe albuminuria)	MTF, insulin	29.9 0.54 0.44^a^	Absent No LVH
6 ♂, 14 years G4P4	DNA Not available	DM, ↓HDL, ↑TG, ↑cholesterol	MTF	37.8 0.54 0.45^a^	Absent No LVH
7 ♀, 14 years M4P4	Type 2 CGL *BSCL2*	DM, ↓HDL, ↑TG, ↑cholesterol, nephropathy (severe albuminuria), peripheral neuropathy, ↑BP	MTF, acarbose, insulin, ciprofibrate	88.7 0.75 0.49^a^	Clinical LVH
8 ♂, 14 years G5P5	Type 2 CGL *BSCL2*	DM, high HOMA-IR score, ↓HDL, ↑TG, nephropathy (moderate albuminuria)	MTF	65.3 0.57 0.41	Absent LVH
9 ♀, 25 years M5P5	Type 1 CGL *AGPAT2*	DM, ↓HDL, ↑TG, ↑cholesterol, ↑BP, nephropathy (severe albuminuria), peripheral neuropathy	MTF, insulin, ciprofibrate	154.0* 0.64 0.47^a^	Clinical/ Advanced LVH
10 ♀, 30 years M5P5	DNA Not available	DM, ↓HDL-c, ↑TG, ↑BP, nephropathy (severe albuminuria)	MTF, insulin, losartan	88.6* 0.60 0.42	Incipient No LVH

CGL, congenital generalized lipodystrophy; CAN, cardiovascular autonomic neuropathy; HDL, high-density lipoprotein; HOMA-IR, homeostasis model assessment-insulin resistance; LVH, left ventricular hypertrophy; LVMI, left ventricular mass index; TG, triglycerides; BP, blood pressure; DM, diabetes mellitus; MTF, metformin

* g/m^2^

^a^cQT prolongation

Doppler sonography of the carotid arteries showed that CGL patients had a greater thickening of right and left cIMT, however no carotid obstruction was observed in CGL. The cQT interval presented longest duration in CGL patients versus healthy group. Five patients with CGL (50%) presented cQT interval prolongation versus one patient (5) in the healthy group (p = 0.009). Table 2 shows the values of left ventricular dimensions, cIMT and cQT interval in the groups.

**Table 2:** Echocardiographic, electrocardiographic and carotid Doppler sonography parameters in patients with congenital generalized lipodystrophy and healthy individuals (n = 30)

Variable	CGL (n = 10)	Healthy group (n = 20)	P
LVH (%)	40 (4)	0	*0.008*
LVMI children and adolescents (g/m^2.7^)	41.4 (29.2; 89.0) n = 8	28.5 (17.1; 37.2) n = 16	*0.001*
LVMI adults (g/m^2^)	121.9 (89.0; 154.6) n = 2	50.4 (41.0; 58.5) n = 4	0.064
Total LVMI (g/m^2.7^ or g/m^2^)	54.5 (29.2; 154.6)	29.6 (17.1; 58.5)	*0.002*
LVDD (mm)	4 (34; 52)	43 (33; 52)	0.791
LVSD (mm)	26 (20; 35)	26 (19; 36)	0.507
IVS (mm)	8 (7; 12)	6 (3; 9)	*0.000*
LVPW (mm)	10 (6; 13)	6 (4; 9)	*0.000*
Ejection fraction (%)	70 (59; 76)	72 (58; 82)	0.279
cIMT (mm)	0.59 (0.53; 0.75)	0.52 (0.38; 0.60)	*0.001*
Right cIMT (mm)	0.60 (0.50; 0.85)	0.50 (0.33; 0.60)	*0.007*
Left cIMT (mm)	0.61 (0.48; 0.65)	0.51 (0.40; 0.66)	*0.010*
cQT interval (s)	0.43 (0.41; 0.49)	0.41 (0.38–0.44)	*0.007*
cQT interval prolongation (%)	50 (5)	5 (1)	*0.009*

Italic values indicate significance of P value (P < 0.05)

CGL, congenital generalized lipodystrophy; cIMT, carotid intimal mean thickness; cQT, corrected QT interval; LVH, left ventricular hypertrophy; LVMI, left ventricular mass index; LVDD, left ventricular diastolic diameter; LVSD, left ventricular systolic diameter; IVS, interventricular septum; LVPW, left ventricular posterior wall

We observed impairment of autonomic modulation in CGL patients with LVH. The high-frequency component of the HRV (parasympathetic control) was lower in LVH patients (235 Hz; 55–603) versus no LVH patients (905 Hz; 147–1840); p = 0.033. Table 3 shows an evaluation of clinical and metabolic parameters and an assessment of cardiovascular autonomic function in CGL patients according to the presence of LVH.

**Table 3:** Clinical, metabolic and cardiovascular autonomic parameters in patients with CGL subdivided into groups with and without left ventricular hypertrophy (n = 10)

Variables	LVH (n = 4)	No LVH (n = 6)	P
Female, % (n)	14 (6; 25)	10 (7; 30)	0.665
Age (years)	75 (3)	50 (3)	0.571
pBMI (%)	21.7 (18.7; 22.9)	17.9 (16.2; 22.0)	0.086
Systolic BP (mmHg)	129 (110; 175)	118 (90; 145)	0.394
Diastolic BP (mmHg)	84 (50; 109)	76 (65; 90)	0.392
Basal HR (bpm)	83 (72; 96)	95 (76; 109)	0.136
30/15 coefficient	1.04 (0.98; 1.59)	1.20 (1.13; 1.57)	0.199
Valsalvacoefficient	1.33 (1.15; 1.82)	1.64 (1.29; 2.34)	0.201
E/I coefficient	1.17 (1.09; 1.46)	1.35 (1.27; 1.57)	0.135
Reduction in SBP (mmHg)	11 (6; 22)	3.5 (0; 12)	0.087
Component of very low frequency (Hz)	350 (299; 694)	652 (88; 4250)	0.670
Component of low frequency (Hz)	316 (139; 756)	482 (158; 4294)	0.670
Component of high frequency (Hz)	235 (55; 603)	905 (147; 1840)	*0.033*
Total amplitude spectrum (Hz)	898 (501; 2054)	2389 (621; 9691)	0.136
LF/HF ratio	1.45 (1.18; 1.69)	0.42 (0.12; 3.7)	0.285

Italic values indicate significance of P value (P < 0.05)

CGL, congenital generalized lipodystrophy; LVH, left ventricular hypertrophy; BMI, body mass index; HR, heart rate; BP, blood pressure; SBP, systolic blood pressure; LF/HF, low frequency/high frequency ratio

### Correlations between clinical, metabolic, and cardiovascular variables and autonomic tests

Correlations between clinical, metabolic, cardiovascular parameters and autonomic tests in our sample (n = 30) were described in Table 4. After adjustments by HOMA-IR, A1c, and systolic and diastolic BP, the association between LVMI and LF (p = 0.011), IVS and LF (p = 0.007), and cIMT and leptin (p < 0.001) were maintained.

**Table 4:** Correlations between clinical, metabolic, and cardiovascular parameters and autonomic tests in patients with congenital generalized lipodystrophy and healthy individuals (n = 30)

n = 30	LVMI	IVS	cIMT	cQT
SBP drop				
r	0.450	0.545	0.437	0.559
p	0.012*	0.002*	0.016*	0.001*
30/15				
r	− 0.509	− 0.448	− 0.332	− 0.495
p	0.004*	0.013*	0.073	0.005*
Valsava				
r	− 0.457	− 0.367	− 0.399	− 0.364
p	0.011*	0.046*	0.029*	0.048*
E/I				
r	− 0.430	− 0.453	− 0.245	− 0.430
p	0.016*	0.012*	0.192	0.019*
VLF				
r	− 0.420	− 0.501	− 0.030	− 0.224
p	0.021*	0.005*	0.873	0.233
LF				
r	− 0.570	− 0.478	− 0.082	− 0.063
p	0.001*	0.007*	0.666	0.741
HF				
r	− 0.691	− 0.593	− 0.396	− 0.375
p	0.000*	0.000*	0.030*	0.041*
LF/HF				
r	0.468	0.283	0.465	0.422
p	0.009*	0.129	0.010*	0.020*
TAS				
r	− 0.650	− 0.415	− 0.180	− 0.286
p	0.000*	0.023*	0.340	0.125
Leptin				
r	− 0.114	− 0.542	− 0.683	− 0.200
p	0.550	0.002*	0.000*	0.288
Triglycerides				
r	0.476	0.395	0.548	0.465
p	0.008*	0.031*	0.002*	0.010*
SBP				
r	0.617	0.594	0.380	0.438
p	0.000*	0.000*	0.038*	0.015*
DBP				
r	0.531	0.419	0.420	0.467
p	0.003*	0.021*	0.021*	0.009*
A1c				
r	0.419	0.372	0.460	0.436
p	0.021*	0.043*	0.011*	0.016*
HOMA-IR				
r	0.789	0.438	0.670	0.676
p	0.000*	0.016*	0.000*	0.000*

SBP, systolic blood pressure; DBP, diastolic blood pressure; 30/15, 30/15 coefficient; E/I, E/I coefficient; VLF, very low frequency component; LF, low frequency component; HF, high frequency component; TAS, total amplitude spectrum; LVMI, left ventricular mass index; IVS, interventricular septum; cIMT, carotid intimal media thickness; cQT, corrected QT interval; A1c, glycated hemoglobin

**Fig. 1:**
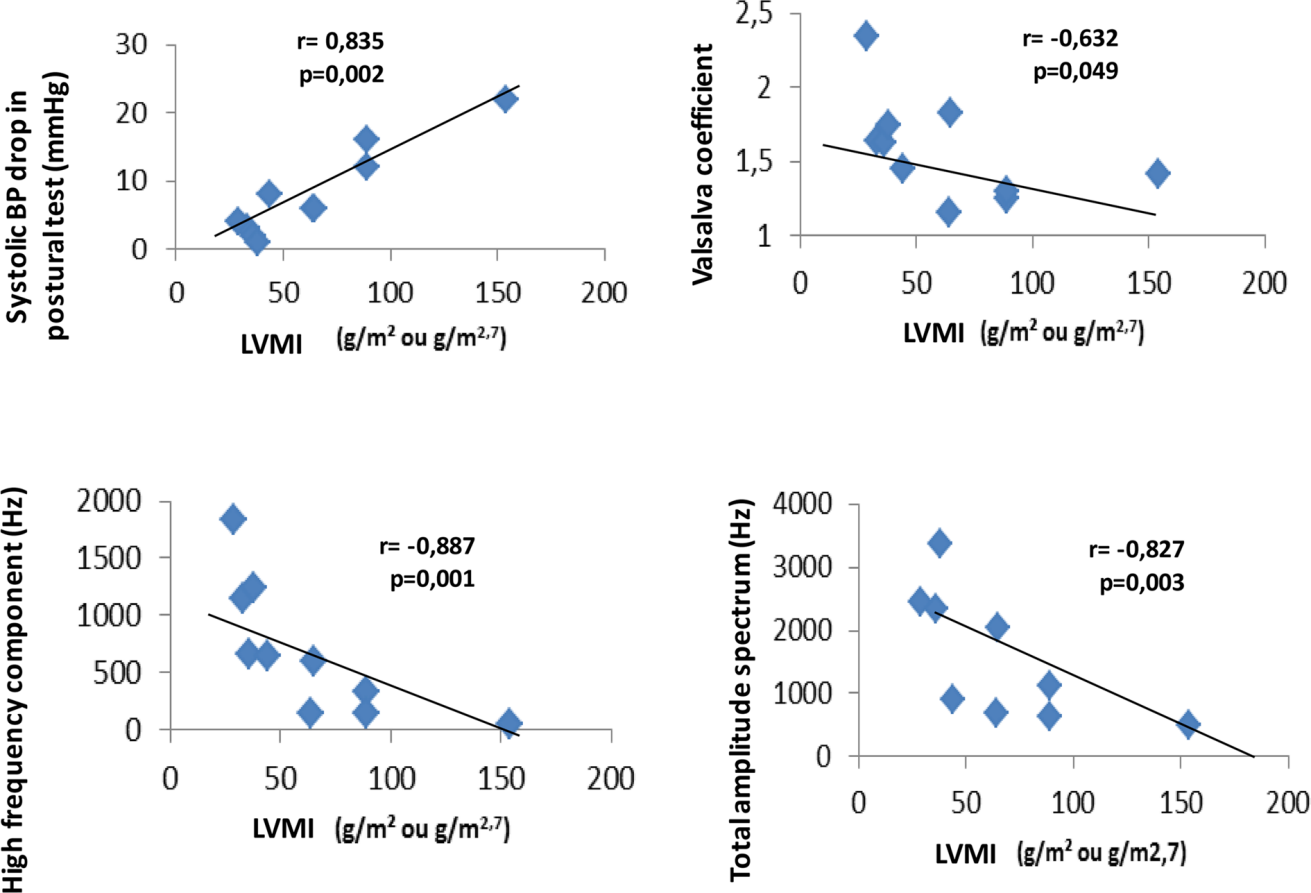
Correlations between the left ventricular mass index and cardiac autonomic neuropathy parameters (n = 10). LVMI, left ventricular mass index

**Fig. 2:**
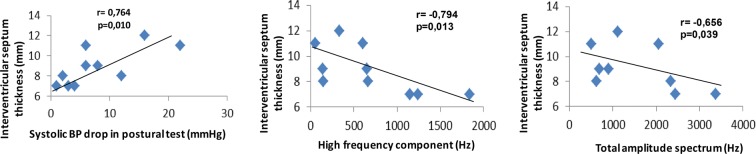
Correlations between the intervetricular septum (IVS) thickness and cardiac autonomic neuropathy parameters (n = 10)

**Fig. 3:**
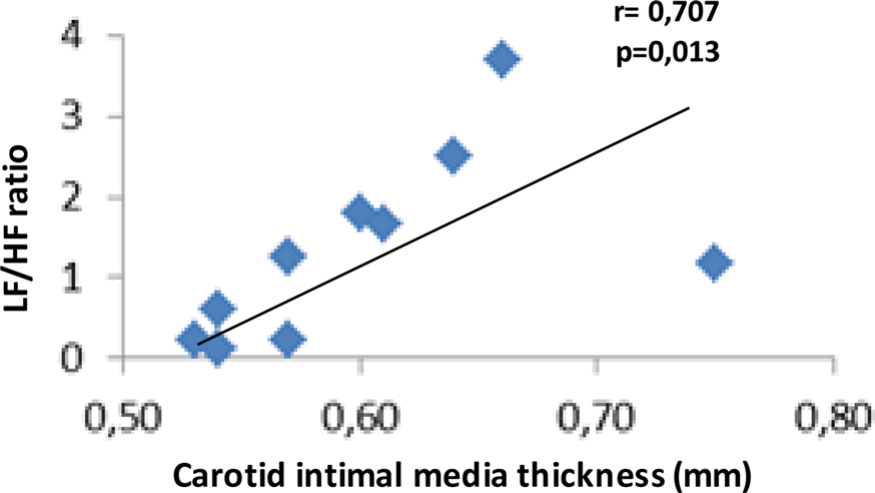
Correlations between the carotid intimal media thickness (cIMT) and cardiac autonomic neuropathy parameters (n = 10). LF/HF, low frequency/high frequency

In the patients with CGL (n = 10), the LVMI correlated positively with systolic blood pressure (BP) drop in the postural test (r = 0.835, p = 0.002), and inversely with Valsalva coefficient (r = − 0.632, p = 0.049), HF component (r = − 0.887, p = 0.001), and TAS (r = − 0.827, p = 0.003) (Fig. 1). The IVS thickness presented positive correlation with systolic BP drop in the postural test (r = 0.764, p = 0.010), and inverse correlation with HF component (r = − 0.794, p = 0.013) and TAS (r = − 0.656, p = 0.039) (Fig. 2). There was a positive correlation between cIMT and LF/HF ratio (r = 0.707; p = 0.022) (Fig. 3). The QTc interval did not correlate with the HRV parameters. After the Bootstrap reassembly technique was applied, LMVI and HF component (IC95% − 1.000; − 0.553), LVMI and TAS (IC95% − 1.000; − 0.012), and IVS and HF component (IC95% − 1.000; − 0.371) maintained the correlation. Between CGL patients, we observed no correlation between cardiovascular parameters (LVMI, IVS, cIMT, and QTc) and baseline HR values and systolic and diastolic BP levels (Additional file 3).

## Discussion

We previously reported a high prevalence of CAN (40%) in young patients with CGL who were evaluated using a gold-standard method, compared to a group of T1DM patients (5%) and healthy subjects (no case of CAN). We observed a significant reduction in CAN parameters (E/I, VLF, LF and HF) in CGL cases vs. type 1 diabetes. In addition, in CGL group there was an association between leptin and 30/15 coefficient, suggesting the metabolic abnormalities observed in CGL may be involved in early CAN development [6].

In the present study, we observed a high frequency of morphologic and electric cardiovascular manifestations in CGL patients, including concentric LVH, carotid atherosclerosis, and cQT prolongation. These abnormalities were observed in young patients, ranging from 7 to 30 years (median 14 years). Most of the patients presented BSCL2 mutation, insulin resistance, and hypertriglyceridemia.

Interestingly, the majority of patients with LVH met criteria for clinical CAN, and we found an association between the LV measurements (LVMI and IVS thickness) and components of the HRV spectral analysis, reflecting parasympathetic and sympathetic autonomic modulation commitment. In diabetic patients, high evidence studies found an association between CAN and LVH [15]. So, by analogy, we could speculate this also may occur in CGL patients. Longitudinal studies where CGL patients were accomplished since young ages could demonstrate a temporal relation between autonomic changes and myocardial abnormalities development.

We know there is biological plausibility to suggest this association. As previously demonstrated, the vagal component is the primary protective mechanism that regulates heart rate, systolic volume, and blood pressure. A reduction of the parasympathetic component is associated with increased systolic blood pressure and LV wall stress, with subsequent LVH, increased the risk of heart failure and cardiovascular disease [16–18]. The predominance of sympathetic tone observed in the early stages of the CAN may promote LVH [18]. The increased sympathetic activity on the myocardium is associated with increased mitochondrial oxidative stress, apoptosis and tissue injury [19, 20]. In experimental studies, autonomic dysfunction promoted the exchange of glucose energetic substrate utilization for free fatty acid (FFA), contributing to the appearance of mitochondrial decoupling, functional and structural ventricular abnormalities and cardiomyopathy [21]. Therefore, autonomic dysfunction could be one of the pathophysiological mechanisms that explain the higher prevalence of LVH in CGL group.

Despite the high prevalence of LVH in this study, no patient presented LV systolic dysfunction. The low age of the patients in this series may explain these results and we believe these parameters would worsen at more advanced ages. In addition, normal resting echocardiographic findings do not entirely exclude the diagnosis of systolic dysfunction. Tissue Doppler imaging during exercise and two-dimensional speckle tracking echocardiography technique present greater diagnostic sensitivity for the earlier stages of ventricular dysfunction [22].

Metabolic abnormalities presented in CGL patients, including insulin resistance, also contribute to the establishment of cardiovascular manifestations. Studies with magnetic resonance imaging showed that insulin resistance is independently associated with LV mass [23]. The insulin resistance is associated with down-regulation of GLUT-4 expression in several cells, including cardiomyocytes, resulting in a reduction in glucose utilization as an energy substrate for the cardiac muscle due to a decrease in glycolysis rate and glucose oxidation, with a consequent increase in the use of FFA as an energetic source in the myocardium [22].

The severe insulin resistance and hyperlipidemia, especially hypertriglyceridemia, observed in patients with CGL increase the oxidation of FFA by cardiomyocytes, a process that requires high levels of oxygen when compared to glucose oxidation, causing relative ischemia on the myocardium. In the presence of ischemia, aerobic glycolysis is replaced by anaerobic glycolysis with accumulation of lactate and acid metabolites. These alterations impair calcium homeostasis and myocardial contraction, a condition known as lipotoxicity [22]. Besides, increased FFA uptake promotes lipid accumulation in cardiomyocytes. Rijzewijk et al. demonstrated the myocardial content of triglycerides evaluated by MRI spectroscopy is increased in patients with type 2 diabetes mellitus (T2DM) and is associated with LV diastolic dysfunction, regardless of age, BMI and visceral fat [24].

We also highlight physiological epicardial adipose tissue (EAT) found reduced or absent in patients with CGL [5, 25]. Evidence suggests that EAT protects cardiomyocytes against the influx of high free fatty acids (FFA) levels and lipotoxicity. Besides, studies have confirmed that EAT secrets adipokines (e.g., adiponectin or leptin) that affect cardiomyocytes function via paracrine and by secretion into the coronary vasa vasorum or coronary circulation. These findings suggest that a local cross-talk between cardiomyocytes and EAT determines many aspects of myocardial biology, cardiac function, and coronary atherosclerosis [26].

Despite the reduction or absence of EAT, Nelson et al. [5]. showed a high triglyceride content in the cardiomyocytes of patients with lipodystrophy (6 congenital and 1 acquired) compared to a matched control group for age, gender, and BMI. Mora et al. demonstrate that leptin affects the myocardial content of triglycerides, acting at central level. Leptin regulates selectively the cardiac expression of PPARβ/δ, contributing to regulate the cardiac triglycerides accumulation in rats, independently of its effects on body weight [27]. Additional studies to evaluation of EAT and intra-myocardial fat content through echocardiogram or MRI, respectively, may contribute to elucidate these associations.

In our previous study [6], we discussed that leptin may be involved in the development of CAN, a condition known to be associated with LVH. Thus, we speculate that in addition to its effects in the autonomic function, hypoleptinemia also may have a role in cardiomyopathy of the CGL patients by modulation of the myocardial content of triglycerides, acting at local and central level. Therefore, we believe the absence of EAT and hypoleptinemia would justify the development of cardiac steatosis, electric remodulation, and LVH.

In experimental studies, the injection of leptin in ob/ob mice has showed a significant reduction in the size of cardiac myocytes, demonstrating an anti-hypertrophic potential of leptin on cardiac cells [28, 29]. Replacement of metreleptin in CGL patients may have a beneficial role in preventing these cardiovascular manifestations. But prospective clinical trials are needed to demonstrate stronger evidence about these speculations.

Another possible explanation for LVH observed in the context of CGL would be the direct action of hyperinsulinemia per se on cardiomyocytes. Insulin can bind to IGF-1 receptors found in abundance in the myocardium, which is responsible for mediating cell growth and differentiation [30]. However, the long-term effect of severe hyperinsulinemia on cardiac function is still poorly understood. In fact, in other severe chronic forms of insulin resistance, such as in the mutations of the insulin receptor, LVH or other manifestations of cardiomyopathy are not frequently encountered, raising questions about the direct impact of hyperinsulinemia on the genesis of associated cardiomyopathy to CGL [4].

In our study, we observed a higher baseline HR in patients with CGL, possibly reflecting the predominance of sympathetic autonomic activity due to the impairment of the parasympathetic modulation. Many studies have shown that HR increase is independently associated with increased cardiovascular and all-cause mortality. In patients with LVH, resting tachycardia contributes to an increased risk of coronary atherosclerotic events [31–33]. Besides, although this study founds no correlation between BP and LVMI, IVS, and LV posterior wall among CGL patients, it is possible that the increase in BP also contributes for LVH development.

In this series, we observed no hypertrophic cardiomyopathy. Rêgo et al. showed a high prevalence of LVH in the echocardiogram (50%) in a series of 22 patients with CGL (mean age 22.4 years) followed in Brazil, and no case of hypertrophic cardiomyopathy was found [3]. Although the first reports define cardiac involvement in CGL as hypertrophic cardiomyopathy, the classic concept of this condition does not characterize the cardiomyopathy pattern observed in these patients. Hypertrophic cardiomyopathy has characteristics typical of the histopathological examination that corresponds to the association of hypertrophy, myocardial fiber derangement and fibrosis [34], unlike the one observed in lipodystrophy, in which no rearrangement of the myocardial fibers is observed [35].

Interestingly, we found an inverse association between leptinemia and cIMT even after adjustment by HOMA, A1c and BP. Physiologically, leptin is suggested to be an important factor in the maintenance of vascular homoeostasis and wall integrity [36]. Leptin induces nitric oxide synthesis and stimulates coronary artery vasodilatation in humans [37]. Nitric oxide has a crucial role in reducing platelet adhesion and vascular smooth muscle cell proliferation [38]. Besides, experimental studies showed leptin promotes recruitment of beneficial vascular endothelial progenitor cells into the vasculature and the injection of leptin in ob/ob mice has demonstrated a significant reduction in wall thickness [28, 29].

In our study, there was an increase in cIMT in patients with CGL, suggesting pre-clinical atherosclerosis. No patient had presented any cardiovascular event until the end of this research protocol. However, later, a female patient (case 9), presented acute myocardial infarction at 30 years and coronary angiography demonstrated triarterial lesion. She was submitted to coronary angioplasty (data not published). These findings corroborate the need for more aggressive strategies to control the cardiovascular risk factors in these patients. In addition, early routine screening to detect atherosclerotic disease through non-invasive tests, as cIMT, could be an interesting option. The cIMT measurement is a readily available, non-invasive technique and presents a good correlation with histological findings in the evaluation and detection of pre-clinical lesions of the arterial wall and may be useful in evaluating the cardiovascular risk of these young patients [39]. Coronary calcium score and MRI also could be used. However, these techniques are costly and are not widely available.

We observed a high frequency of prolonged QT interval among patients with CGL. Long QT interval has been previously described in these patients, especially in those with subtypes 2 and 4 of the syndrome [2]. The pathogenesis of acquired long QT is multifactorial and includes imbalance of cardiac sympathetic innervation, metabolic and intrinsic electrolyte myocardial changes, LVH, and CAD [40, 41]. QT prolongation is an independent predictor of all-cause and cardiovascular mortality [42, 43], and in our understanding should be systematically investigated in CGL patients given their high prevalence and increased risk of arrhythmias and death.

The main limitation of this study was the sample size of the CGL group. In an attempt to minimize this limitation, we use the Bootstrap resampling technique, which consists of a statistical computational tool that assumes the simulation of several sample scenarios from a single sample and in this way try to construct a sample space and to make inferences about the parameter of interest. Also, the patients performed the exams in the pharmacological treatment of metabolic abnormalities. However, none of them were using drugs with direct action on the ANS or with known effects on the cardiovascular autonomic tests.

Another limitation of this research was the lack of the molecular study in some cases of CGL, which compromised the analyzes in the subgroups with CGL subtypes 1 and 2 and to infer about molecular mechanisms involved. Seven patients had genetic analysis. Between CGL2 cases (*BSCL* mutation; n = 5), 3 patients had LVH at early ages: 7 year, (case 1), 14 yr (case 7), and 14 year (case 8). Between CGL1 patients (*AGPAT2* mutation; n = 2), 1 patient had LVH (case 9; 24 year). Another CGL1 patient had not LVH, but she was very young (case 2; 7 year). As previously described [2], these findings suggest cardiovascular manifestations, in addition to metabolic abnormalities, are earlier in individuals with CGL2.

We emphasize the cardiovascular autonomic reflex tests followed a standard protocol considered the gold standard for the diagnosis of the CAN. The sample of CGL patients from this study was characterized by a wide age range, from 7 to 30 years, including prepubertal children, adolescents, and adults. Despite the small sample size of this study, this distribution allowed to evaluate the different profiles of clinical manifestations and metabolic complications of CGL, allowing a better understanding of this rare disease that has been considered an important biological model for the study of the role of adipose tissue as an endocrine organ. Besides, it is the first study to demonstrate an association between CAN and LVH in patients with CGL.

## Conclusion

In conclusion, the association between increased LV mass and parameters of HRV provides possible speculations about the involvement of CAN in the pathophysiology of the cardiac complications, including LVH, in CGL patients. Future studies in different CGL populations may prove the consistency of our findings.

## Additional files

**Additional file 1.** Cardiovascular autonomic reflex tests.

**Additional file 2.** Clinical, biochemical and CAN parameters in patients with congenital generalized lipodystrophy and healthy individuals (n = 30).

**Additional file 3.** Correlations between clinical and cardiovascular variables and autonomic tests in patients with CGL (n = 10).

## Data Availability

The datasets used and/or analyzed during the current study are not publicly available due risk that participants might be identifiable is considered non-negligible (indirect identifiers: age, sex, rare disease anthropometry measures, small denominator and numerators) but are available from the corresponding author on reasonable request.

## Abbreviations

ACR: albumin/creatinine ratio
BMI: body mass index
BP: blood pressure
BRAZLIPO: Brazilian Group for the Study of Inherited and Acquired Lipodystrophies
CAD: coronary artery disease
CAN: cardiac autonomic neuropathy
CGL: congenital generalized lipodystrophy
cIMT: carotid intimal media thickness
cQT interval: corrected QT interval
EAT: epicardial adipose tissue
EF: ejection fraction
FFA: free fatty acids
GFR: glomerular filtration rate
HbA1c: glycated hemoglobin
HDL: high density lipoprotein
HF: high frequency
HOMA-IR: homeostasis model assessment-insulin resistance
HRV: heart rate variability
IGF-1: insulin growth factor 1
IVS: interventricular septum thickness
LF: low frequency
LV: left ventricle
LVH: left ventricular hypertrophy
LVMI: left ventricular mass index
NDS: neuropathy disability score
RPLV: relative thickness of the posterior wall of left ventricle
TAS: total amplitude spectrum
TSS: neuropathy total symptom score
T2DM: type 2 diabetes mellitus
us-CRP: ultra-sensitive C reactive protein
VLF: very low frequency
