# Supplementary material for: Association between cardiovascular autonomic neuropathy and left ventricular hypertrophy in young patients with congenital generalized lipodystrophy
Source: Diabetol Metab Syndr. 2019 Jul 1;11:53. doi: 10.1186/s13098-019-0444-8 (PMC6604128; doi:10.1186/s13098-019-0444-8)
Supplement: Supplementary file 1 — Additional file 1. Cardiovascular autonomic reflex tests. [file 13098_2019_444_MOESM1_ESM.docx]

**Additional file**

**Additional file S1: Cardiovascular Autonomic Reflex Tests**

Individuals were evaluated in fasting state during the morning. Capillary glycemia was determined before the beginning of the test and had to be in the range of 70 and 250 mg/dL. The patients were asked not to inject rapid-acting insulin at least two hours before the beginning of the tests, to avoid the use of caffeine for at least eight hours before the test and not vigorously to exercise for 24 hours before the evaluation. Volunteers presenting with a fever (temperature ≥ 37.8 °C) in the previous two days, considerable emotional stress in the previous day or hypoglycemia at 8 hours before the tests were asked to postpone testing.

The tests were started at 15 minutes after resting with the patient lying down and a head inclination of 30 degrees. After this time interval, a 300-second electrocardiogram was performed. The electrocardiogram was analyzed using a mathematical algorithm (Fourier transformation) and expressed in a diagram with amplitude oscillations (cardiac frequency fluctuations per second) versus the cardiac frequency (hertz) (spectral analysis). The total amplitude of HRV or total amplitude spectrum (TAS) comprised three bands: (1) very low-frequency component or VLF (0.01-0.04 Hz), (2) low-frequency component or LF (0.04- 0.15 Hz) and (3) high-frequency component or HF (0.15- 0.5 Hz). The relation between LF/HF was calculated, reflecting the sympathetic-vagal balance.

After the evaluation of the HRV spectral, cardiovascular autonomic reflexes tests were performed. A resting period of 1 minute was maintained between tests to prevent influences from previous tests.

1. In-depth breathing test (E/I coefficient): during the electrocardiogram, the volunteer has performed deep inspiration and expiration with at least a 5-second duration each. The duration of each respiratory cycle was signaled from the volunteer to the researcher. The E/I coefficient was obtained by dividing the most extended RR interval [minimum heart rate (HR) during expiration] by the shortest RR interval (maximal HR during inspiration). Each respiratory cycle was repeated three times, and the best ratio obtained was considered to be the final result.
2. Valsalva maneuver: during the electrocardiogram, the volunteer performed a breathing exercise to maintain a pressure of 40 mmHg that was evaluated using a manometer for 15 seconds. At approximately 14 seconds, a maximal physiologic tachycardia was expected to occur. After this exercise, deep breathing was interrupted, and the electrocardiogram register as maintained for 45 seconds, while physiologic bradycardia was expected to occur. The Valsalva coefficient was obtained according to the relation between the longest (minimal HR) and shortest (maximal HR) RR intervals. The occurrence of a facial flushing, plethora and cervical veins engorgement indicated that the test was adequately performed. The best out of two results was considered the definitive result.
3. Orthostatic test (30/15 coefficient): after resting in the supine position, the volunteer had to remain in an orthostatic position, and the relation between the RR interval corresponding to the maximal bradycardia around the 30th heartbeat and the maximal tachycardia around the 15th heartbeat after the orthostatic position was considered to be the final result.
4. Orthostatic or postural hypotension test: after a 30 minutes rest in the supine position, the arterial blood pressure was evaluated and compared with the pressure at 3 minutes after the beginning of the orthostatic position. A drop equal to or higher than 20 mmHg in the systolic blood pressure was considered altered.

Reference: Spallone V, Bellavere F, Scionti L, Maule S, Quadri R, Bax G, et al. Recommendations for the use of cardiovascular tests in diagnosing diabetic autonomic neuropathy. NutrMetab Cardiovasc Dis. 2011 Jan;21(1):69-78. PubMed PMID: 21247746. Epub 2011/01/21. eng.
