# Supplementary material for: Association between cardiovascular autonomic neuropathy and left ventricular hypertrophy in young patients with congenital generalized lipodystrophy
Source: Diabetol Metab Syndr. 2019 Jul 1;11:53. doi: 10.1186/s13098-019-0444-8 (PMC6604128; doi:10.1186/s13098-019-0444-8)
Supplement: Supplementary file 2 — Additional file 2. Clinical, biochemical and CAN parameters in patients with congenital generalized lipodystrophy and healthy individuals (n = 30). [file 13098_2019_444_MOESM2_ESM.docx]

**Additional file S2: Clinical, biochemical and CAN parameters in patients with congenital generalized lipodystrophy and healthy individuals (n=30)**

| **Variables** | **CGL**  **(n=10)** | **Healthy group**  **(n=20)** | **P** |
| --- | --- | --- | --- |
| **Female, % (n)** | 60 (6) | 60 (12) | 1.000 |
| **Age (years)** | 12 (7; 30) | 12 (7; 31) | 0.642 |
| **pBMI (%) children and adolescents** | 62 (41; 94) n=8 | 52 (10; 82) n=16 | 0.100 |
| **BMI (Kg/m²) adults** | 22.3 (22.0; 22.7) n=2 | 23.7 (22.5; 24.8) n=4 | 0.165 |
| **Age group** | Pre-pubertal: 10 (1)  Pubertal: 70 (7)  Adult: 20 (2) | Pre-pubertal: 20 (4)  Pubertal: 60 (12)  Adult: 20 (4) | 0.508 |
| **Basal HR (bpm)** | 90 (72; 109) | 71 (53; 94) | **0.006** |
| **Systolic BP (mmHg)** | 123 (90; 175) | 104 (80; 113) | **0.001** |
| **Diastolic BP (mmHg)** | 78 (50; 109) | 66 (60; 80) | **0.006** |
| **Diabetes Mellitus, % (n)** | 70 (7) | 0 | **0.000** |
| **Diabetes duration (years)** | 8 (1; 14) | 0 | 0.260 |
| **Glycated hemoglobin (mmol/mol)** | 55 (25; 109) | 33 (22; 40) | **0.006** |
| **Glycated hemoglobin (%)** | 7.2 (4.4; 12.1) | 5.2 (4.2; 5.8) | **0.006** |
| **Nephropathy, % (n)** | 60 (6) | 0 | **0.000** |
| **Use ofinsulin, % (n)** | 40 (4) | 0 | **0.008** |
| **Fasting plasma glucose (mmol/l)** | 5.4 (3.8; 13,8) | 4.5 (4.7; 5.2) | **0.045** |
| **Total cholesterol (mmol/l)** | 3.5 (2.5; 20.0) | 4.0 (2.1; 5.1) | 0.644 |
| **HDL-cholesterol (mmol/l)** | 0.8 (0.6; 1.3) | 1.4 (1.2; 2.2) | **0.000** |
| **LDL-cholesterol (mmol/l)** | 2.1 (1.2; 4.0) | 2.2 (1.1; 3.7) | 0.611 |
| **Triglycerides (mmol/l)** | 1.3 (1.0; 80.4) | 0.8 (0.5; 1.7) | **0.001** |
| **Leptin (ng/mL)** | 1.1 (0.8; 1.7) | 4.9 (1.3; 33.0) | **0.000** |
| **PCRus** | 0.14 (0.10; 1.99) | 0.10 (0.10; 1.00) | **0.022** |
| **Basal insulin (mUI/mL)** | 22,8 (6,7; 102,0) | 9,8 (2,8; 13,4) | **0.000** |
| **HOMA-IR** | 6,8 (1,2; 15,1) | 2,2 (0,5; 2,8) | **0.000** |
| **ACR (mg/g)** | 85.3 (3.1; 5535.0) | 5.9 (2.5; 24.9) | **0.004** |
| **Clinical CAN % (n)** | 40 (4) | 0 | **0.008** |
| **Incipient CAN % (n)** | 10 (1) | 0 | 0.333 |
| **30/15 coefficient** | 1.19 (0.98; 1.59) | 1.55 (2.26; 2.02) | **0.001** |
| **E/I coefficient** | 1.33 (1.09; 1.57) | 1.60 (1.23; 2.23) | **0.001** |
| **Valsalva coefficient** | 1.54 (1.15; 2.34) | 1.74 (1.50; 2.42) | **0.039** |
| **Reduction in SBP > 10 mmHg % (n)** | 30 (3) | 5 (1) | 0.095 |
| **Reduction in SBP > 20 mmHg % (n)** | 10 (1) | 0 | 0.333 |
| **Component of very low frequency (Hz)** | 383 (88; 4250) | 1988 (688; 18341) | **0.002** |
| **Component of low frequency (Hz)** | 329 (139; 2525) | 1916 (343; 5580) | **0.001** |
| **Component of high frequency (Hz)** | 627 (55; 1840) | 2993 (270; 9969) | **0.001** |
| **Total amplitude spectrum (Hz)** | 1582 (501;9691) | 7125 (1853; 24788) | **0.000** |
| **LF/HF ratio** | 1.21 (0.12; 3.70) | 0.58 (0.26; 1.70) | 0.454 |

Note: Adapted from Ponte et al (2018).^4^

**Abbreviations:** CGL, congenital generalized lipodystrophy; BMI, body mass index; HR, heart rate, BP, blood pressure; us-CRP, ultra-sensitive C reactive protein; ACR, albumin/creatinine ratio; CAN, cardiac autonomic neuropathy; SBP, systolic blood pressure; LF/HF, low frequency/high frequency ratio.
