# Supplementary material for: Association between cardiovascular autonomic neuropathy and left ventricular hypertrophy in young patients with congenital generalized lipodystrophy
Source: Diabetol Metab Syndr. 2019 Jul 1;11:53. doi: 10.1186/s13098-019-0444-8 (PMC6604128; doi:10.1186/s13098-019-0444-8)
Supplement: Supplementary file 3 — Additional file 3. Correlations between clinical and cardiovascular variables and autonomic tests in patients with CGL (n = 10). [file 13098_2019_444_MOESM3_ESM.docx]

**Additional file S3: Correlations between clinical and cardiovascular variables and autonomic tests in patients with CGL (n=10)**

| n=10 | | Age | BMI | Basal HR | SBP | DBP |
| --- | --- | --- | --- | --- | --- | --- |
| Reduction in SBP | r | 0.530 | 0.581 | 0.204 | 0.443 | 0.570 |
|  | p | 0.114 | 0.078 | 0.571 | 0.198 | 0.085 |
| 30/15 | r | -0.092 | -0.018 | 0.115 | -0.406 | -0.579 |
|  | p | 0.799 | 0.959 | 0.749 | 0.244 | 0.079 |
| Valsalva | r | -0.036 | -0.012 | 0.182 | 0.042 | -0.620 |
|  | p | 0.919 | 0.973 | 0.614 | 0.907 | 0.055 |
| E/I | R | -0.270 | -0.384 | -0.103 | -0.406 | -0.827 |
|  | P | 0.449 | 0.273 | 0.776 | 0.244 | 0.003 |
| VLF | R | -0.227 | -0.237 | -0.218 | -0.442 | -0.389 |
|  | P | 0.526 | 0.508 | 0.543 | 0.200 | 0.266 |
| LF | R | -0.233 | -0.213 | -0.376 | -0.454 | -0.541 |
|  | P | 0.515 | 0.553 | 0.283 | 0.186 | 0.106 |
| HF | R | -0.326 | -0.469 | 0.133 | -0.430 | 0.369 |
|  | P | 0.357 | 0.171 | 0.712 | 0.214 | 0.293 |
| LVMI | R | 0.697 | 0.072 | -0.271 | 0.474 | 0.582 |
|  | P | 0.030 | 0.018 | 0.448 | 0.166 | 0.077 |
| IVS | R | 0.311 | 0.525 | -0.109 | 0.130 | 0.171 |
|  | P | 0.381 | 0.117 | 0.765 | 0.720 | 0.637 |
| LVPW | R | 0.195 | 0.415 | -0.095 | 0.177 | -0.110 |
|  | P | 0.588 | 0.232 | 0.794 | 0.624 | 0.761 |
| cIMT | R | 0.034 | 0.138 | 0.027 | -0.061 | 0.477 |
|  | P | 0.925 | 0.703 | 0.940 | 0.867 | 0.163 |

LEGEND: CGL: congenital generalized lipodystrophy; SBP: systolic blood pressure; 30/15: 30/15 coefficient; E/I: E/I coefficient; VLF: very low frequency component; LF: low frequency component; HF: high frequency component; LVMI: left ventricular mass index; IVS: interventricular septum; LVPW: left ventricular posterior wall; cIMT: carotid intimal media thickness.
